# Positive responses to stress in the situation of the COVID-19 pandemic (in Russian sample)

**DOI:** 10.1192/j.eurpsy.2022.960

**Published:** 2022-09-01

**Authors:** E. Karacheva, M. Magomed-Eminov, O. Savina, O. Kvasova, O. Magomed-Eminova

**Affiliations:** 1 Moscow State University, Psychology Department, Moscow, Russian Federation; 2 Lomonosov Moscow State University, Department Of Psychological Help And Resocialization, Moscow, Russian Federation

**Keywords:** coping, mental health, positive response

## Abstract

**Introduction:**

The aim of the study was to adapt the Coping Self-efficacy Scale for research Russian population in the situation of the COVID-19 pandemic for researching positive personal resources to overcome peritraumatic COVID-19 distress. To solve this task we also used Impact of Event Scale (Horowitz) and Post-Traumatic Growth Inventory (Tadeshi & Calhoun) - both adapted by M. Magomed-Eminov. These two methods allow us to assess the connection coping self-efficacy with both the traumatic experience and the experience of post-traumatic growth. And to use the results to prevent mental health.

**Objectives:**

342 participants (students and masters; 18,2% male, 81,8% female; age: 20-30 years).

**Methods:**

Russian version of Coping self-efficacy scale developed in Psychological Helping and resocialization Department Lomonosov Moscow State University; Post-Traumatic Growth Inventory – PTGI (Tadeshi & Calhoun), Impact of Event Scale (Horowitz), - both adapted by M. Magomed-Eminov.

**Results:**

Russian version of Coping self-efficacy scale has high reliability-consistency (Cronbach’s α = 0.916). Detected significant correlation between coping self-efficacy and post-traumatic growth (rS = 0,261, p < 0,01) and significant negative correlation between coping self-efficacy and intensity of the impact of stressful events (IES) (rS = - 0,140, p < 0,05).

**Conclusions:**

The obtained results confirmed the high psychometric effectiveness of the Self-efficacy Coping Scale. The connections indicate the existence of positive ways of coping to distress. The results obtained suggest that further research on the positive consequences will expand the repertoire of tools predicted the ability of a modern person to cope with adversity and use experience for deeper involvement of human resources.

**Disclosure:**

No significant relationships.

